# Exploring Midwives' and Nurse‐Midwives' Professional Identity and How Midwifery May Be Best Represented in the Public Realm: A Global Convergent Parallel Mixed‐Methods Study

**DOI:** 10.1111/jan.16696

**Published:** 2024-12-26

**Authors:** Sally Pezaro, Gila Zarbiv, Jude Jones, Mariama Lilei Feika, Laura Fitzgerald, Sanele Lukele, Jacquelyn McMillan‐Bohler, Olivia B. Baloyi, Ksenija Maravic Da Silva, Christine Grant, Lisa Bayliss‐Pratt, Pandora Hardtman

**Affiliations:** ^1^ The Research Centre for Healthcare and Communities Coventry University Coventry UK; ^2^ The University of Notre Dame Fremantle Victoria Australia; ^3^ Department of Obstetrics and Gynecology, Hadassah‐Hebrew University Medical Center and Faculty of Medicine Hebrew University of Jerusalem Jerusalem Israel; ^4^ Global Health Unit NHS Manchester UK; ^5^ Al Khor Hospital – Hamad Medical Corporation Al Dhakira Qatar; ^6^ Jhpiego, An Affiliate of Johns Hopkins University Baltimore Maryland USA; ^7^ Department of Nursing University of Johannesburg Johannesburg South Africa; ^8^ School of Nursing Duke University Durham North Carolina USA; ^9^ School of Nursing and Public Health University of KwaZulu‐Natal Durban South Africa; ^10^ Fatima College of Health Sciences Abu Dhabi UAE; ^11^ Jhpiego Baltimore Maryland USA

**Keywords:** health personnel, midwife, midwifery, midwives, nurses, nursing, occupation, professional identity, science in the arts, workforce

## Abstract

**Aims:**

With respect to midwives and nurse‐midwife populations (1) measure and (2) explore professional identity and (3) explore how the midwifery profession may be best represented in the public realm.

**Design:**

Convergent Parallel Mixed‐Methods Design.

**Methods:**

A web‐based survey was used to collect data from 860 midwives and nurse‐midwives from 102 countries between February and July 2022. Qualitative data were analysed inductively via reflexive thematic analysis. Statistical analysis was performed using SPSS.

**Results:**

Nurse‐midwives had a statistically significant higher professional identity score than midwives. Subthemes included pride and passion in midwifery and nurse‐midwifery; motivational religiosity; celebrating role diversity and a call for the separation of nursing and midwifery. Subthemes outlining barriers to the development of a healthy professional identity related to lack of professional recognition in society and lack of respect from other professions. Subthemes related to the representation of midwifery included (1) imagery (e.g., real midwives) and (2) mediums (e.g., statues). Diminished professional identity and the conflation of midwifery with nursing were negatively associated with the recruitment and retention of midwives.

**Conclusion:**

This is the first study to measure and investigate the professional identity of midwives and nurse‐midwives concomitantly and explore how midwifery may be best represented in the public realm. Public monuments highlighting both the art and science of midwifery along with the separation of midwifery from the nursing profession and enhanced role diversity may boost the professional identity of midwives overall.

**Implications for the Profession:**

Midwives' professional identity, status and recognition in society are key to improved perinatal outcomes, recruitment and retention. Findings will inform interventions designed to enhance the professional identity and public representation of midwives worldwide.

**Impact:**

This research demonstrates midwives diminished professional identity. Findings will be used to bolster the midwifery profession to improve perinatal outcomes, along with the recruitment and retention of midwives to the benefit of childbearing people and their families worldwide.

**Patient or Public Contribution:**

Global webinars were used to engage midwives and nurse‐midwives in shaping the design and direction of this research.

**Reporting Method:**

The Good Reporting of a Mixed Methods Study (GRAMMS) checklist was used to guide reporting.

**Summary**

- What does this paper contribute to the wider global clinical community?
  ○ Foundational new knowledge in relation to how midwives and nurse‐midwives' professional identities compare with one another on a global scale.
  ○ Midwives and nurse‐midwives' perspectives about how midwives should be represented in a context where societal representations and perceptions are influential in shaping professional identities.
  ○ New insights in relation to midwives and nurse‐midwives professional identity, considered essential for optimal clinical outcomes and staff recruitment and retention, on a global scale.

## 1 Introduction

Broadly, a profession can be described as a specialised field of work with its own body of knowledge and ethical guidelines (Freidson 2001), whilst a professional is someone who has the expertise and commitment to practise within their profession or field (e.g., midwifery), typically with a unique professional identity (Cruess, Cruess, and Steinert 2016). Professional identity relates to how people see themselves and is based upon values, motives, experiences, attributes and beliefs specifically in relation to their profession (Rees and Monrouxe 2018). In healthcare, lower levels of professional identity and lack of professional recognition are notably related to poorer standards of care (Traynor and Buus 2016), along with higher levels of burnout and increased turnover intentions (Zhang et al. 2021). As critical members of the global healthcare workforce, midwives currently confront all of these professional challenges (Andina‐Díaz et al. 2024; Cull et al. 2020; Pezaro et al. 2016; Vermeulen et al. 2023). Thus, it is important for research to explore how midwives' professional identities, status and recognition in society may be optimised for improved outcomes, recruitment and retention.

## 2 Background

Whilst the development of a healthy professional identity is considered vital to a variety of healthcare cadres, midwives' distinct professional identity is in jeopardy (Healy, Humphreys, and Kennedy 2017). Global calls aim to strengthen midwives' profile and status, harmonise the profession through a common philosophy, and solidify an overall professional identity for them (Kemp, Maclean, and Moyo 2021). Yet ultimately, in order for midwives to fulfil their essential role and potential in reducing the majority of unacceptable and preventable perinatal deaths worldwide (Renfrew 2021), it is argued that their professional identity must first be clearly instantiated in the public realm (e.g., through public exemplification or representation) (Pezaro, Maher, and Fissell 2022). Indeed, societal representations and perceptions are influential in reciprocally shaping one's professional identity (Pelini 2011). Thus, it will be useful to understand how the profession may be best represented from the perspective of midwives, particularly as a lack of their professional recognition in society has been linked to a range of negative outcomes (Andina‐Díaz et al. 2024; Vermeulen et al. 2023).

In addition to societal influences (Pelini 2011), midwives form their professional identities through a dynamic interplay of education, clinical experience, personal reflection and professional engagement (Cruess, Cruess, and Steinert 2016; Phillips and Hayes 2006). These identities are central to their ability to provide compassionate, competent and ethical care to the individuals and communities they serve (Ellis and Hogard 2020). Yet contextually, educational backgrounds differ both across and within countries, as the International Confederation of Midwives (ICM) outline in detail from country to country (UNFPA 2021). For example, data collected from 80 countries on their midwife education programmes indicate that 33 (41%) offer only direct‐entry programmes, 17 (21%) offer only post‐nursing, five (6%) offer combined nursing and midwifery and 25 (31%) offer both direct‐entry and another type of programme (UNFPA 2021). Thus, in some geographical areas, being a nurse is a prerequisite to becoming a midwife, though, in other parts of the world, this is not the case. Some countries rely on nurses who have instead undertaken additional and specialised training in midwifery. These professionals are often known as ‘nurse‐midwives’ who may practise as part of the ‘wider midwifery workforce’. Yet the term ‘nurse‐midwife’ suggests a duality of roles, as individuals could technically claim membership and form identities within either professional group. Of the 1.9 million members of the wider midwifery workforce around the world (UNFPA 2021), 651,000 (34%) are categorised as midwifery professionals, 477,000 (25%) as midwifery associate professionals, 421,000 (22%) as ‘midwives not further defined’, 285,000 (15%) as nurse‐midwives and 60,000 (3%) as associate nurse‐midwives. Midwives' scope of practice can also vary significantly, where many are not authorised to perform tasks typically considered to be part of the midwife's scope of practice, most commonly in the Americas, European and the Eastern Mediterranean regions, and in high‐income countries (UNFPA 2021). Due to these disparities, it is important to recognise that midwives and nurse‐midwives will not all have the same characteristics and may not all have the same sense of professional unity worldwide.

Global calls look to distinguish between midwives, nurses and nurse‐midwives where applicable (UNFPA 2021). Whilst ‘nurse‐midwifery’ in conjunction with ‘midwifery’ has also been formalised into its own licenced profession (Ettinger 2006), it remains unclear if those who are registered or licenced to practise in this dual role have stronger or weaker professional identities when compared to those solely licenced to practise midwifery. Scoping searches of the literature revealed no published studies in which the levels of professional identity in both midwife and nurse‐midwife populations had been both measured in a global context (Cornett, Palermo, and Ash 2023). Yet, an understanding and measurement of these distinctions in a context where midwives and nurse‐midwives are conflated may be useful to guide the direction of future midwifery workforce initiatives (Ellis and Hogard 2020), particularly with regards to professional identity, societal recognition and status.

## 3 The Study

### 3.1 Research Aims

With respect to midwives and nurse‐midwife populations, the aims of this study were to (1) measure, (2) explore professional identity and (3) explore how the midwifery profession may be best represented in the public realm.

## 4 Materials and Methods

### 4.1 Study Design

We adopted a convergent parallel mixed‐methods design (Creswell and Clark 2017). Mixed methods approaches are particularly useful when tackling complex research topics because they integrate both post‐positivism and interpretivism as philosophical frameworks (Fetters 2016). They also connect both qualitative and quantitative data in such a way that research findings can be meaningfully explained (Dawadi, Shrestha, and Giri 2021). The convergent approach was considered above either an explanatory or exploratory sequential mixed‐methods design as it allowed us to gather both quantitative and qualitative data simultaneously, providing a more holistic and nuanced understanding of the research problem (e.g., the ‘what’ [quantitative] and the ‘why’ [qualitative] together) (Mertens 2023). Lastly, convergent designs give equal weight to both quantitative and qualitative data, avoiding the hierarchy inherent in sequential designs, where one approach informs the other. For this reason, our mixed‐methods approach was equivalently driven by both quantitative and qualitative elements (Moseholm and Fetters 2017), though we recognise that no quantitative data were collected in relation to our third research aim.

**FIGURE 1:**
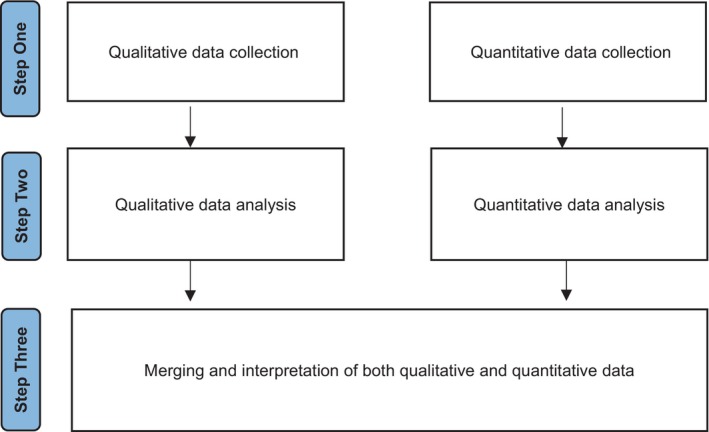
Convergent parallel mixed‐method study design.

The need to transcend local contexts in pursuit of a broader understanding of this worldwide problem directed our global approach. Whilst the collection of quantitative data on a large scale remains a standard approach in research, the collection of qualitative data on a similarly large scale is fast also becoming established as an exciting and flexible choice in the design of research and has previously been used successfully in samples of this nature (Pezaro, Zarbiv et al. 2024). Indeed, challenges have been made to the traditional view of surveys as primarily quantitative instruments paving the way for new and exciting research to harness their potential (e.g., via open text responses) for generating rich and meaningful qualitative data on a large scale (Braun et al. 2021). Considering the above, we used a web‐based survey to collect both qualitative and quantitative data on a large scale and in parallel between February and July 2022. In this separative approach (Moseholm and Fetters 2017), the quantitative and qualitative data analyses were conducted independent of each other prior to a final merging and interpretation of the two strands (Figure 1).

### 4.2 Study Setting and Sampling

Our study setting was global. Accordingly, those licenced/registered to practise either as a midwife or nurse‐midwife anywhere in the world were eligible to participate. No age restrictions were placed on potential participants. Limitations to survey participation included exclusion of potential participants who did not have access to the internet or those who could not read or write in English.

Recruitment commenced on the 14 February 2022 during a global webinar hosted by the Nursing Now Challenge (NNC) and the Midwives in Focus Programme (www.nursingnow.org/midwives‐infocus). The NNC is a global programme working with health employers around the world to create leadership development opportunities for 100,000 nurses and midwives in more than 150 countries. This programme uses social media to develop networks of health professionals learning from each other's experiences and expertise across the world. The ‘Midwives in Focus’ programme falls under the umbrella of the NNC and is an initiative aiming to raise the profile of midwives and midwifery in this context.

Delegates of our recruitment webinar (*n* = 64) joined from 26 countries. Attendees were invited to participate and share recruitment information alongside the survey link with their local, regional and global professional networks. These were also shared via monthly email to all members of the NNC and the Global Alliance for Nursing and Midwifery (GANM). Furthermore, recruitment information was shared via the World Health Organisation's Nursing and Midwifery Community of Practice online platform, Facebook and Twitter (now X) to encourage global participation throughout our data collection period. Follow‐up webinars (*n* = 3) were also held throughout the data collection period to maximise recruitment efforts and maintain global engagement. Once participants offered their consent and confirmed their eligibility to participate via the survey link, they were subsequently granted access to the remainder of the survey.

### 4.3 Quantitative Data Collection

Demographic questions related to participants' age, gender, ethnicity and licensure. Participants' professional Identity was measured using the Professional Identity Scale—Healthcare Students and Professionals (PIS‐HSP) (Liao and Wang 2020). This scale is comprised of four subscales asking questions in relation to ‘professional commitment and devotion’ (6 items), ‘emotional identification and belongingness’ (4 items), ‘professional goals and values’ (4 items) and ‘self‐fulfilment and retention tendency’ (4 items). The items are assessed on a nine‐point Likert scale with 9 indicating extreme agreement and 1 extreme disagreement. The averages of the items represent the final four scores of the four dimensions: the higher the score, the stronger the professional identity a participant holds. Overall professional identity score is measured as an average of all 18 items. The words ‘healthcare professional’ were amended to ‘midwifery/Nurse‐Midwifery’ on the scale to more accurately reflect the professional identity of participants.

### 4.4 Qualitative Data Collection

In order to meet the second aim of this research, participants were subsequently invited to share written reflections on their professional identity. They were then invited to share their written reflections on what might best represent the profession of midwifery in wider society in order to meet the final aim of this research. At the end of the survey, participants were encouraged to explore resources on maintaining individual wellbeing.

### 4.5 Quantitative Data Analysis

For the quantitative element of the survey, 1026 participants were recruited. If more than 10% of the data was missing, the responses were removed from our analyses. A highly significant skew of the statistical data warranted a more conservative approach using non‐parametric Mann–Whitney *U* test to compare midwives with nurse‐midwives across the different dimensions of the professional identity scale. Further, due to multiple tests, an alpha value of *p* < 0.012 was accepted as a statistically significant result.

### 4.6 Qualitative Data Analysis

The qualitative data offered in relation to *a priory* themes of both professional identity and representations of the midwifery profession were analysed inductively via reflexive thematic analysis (Braun and Clarke 2006, 2021). Statements aligned with either professional identity or representations of the midwifery profession were then separated into semantic codes. Codes which corresponded with one another were then assembled in an iterative succession of refinements. They were then arranged into groups which collectively represented frequently linked concepts. Distinct group features which best exemplified the dataset were then drawn upon to develop subthemes under *a priory* themes.

In terms of positionality, the team reflected upon their diversity with direct entry registered midwives, certified nurse‐midwives and psychologists represented. Our team also represented diversity in practice (e.g., clinical, leadership, research, education and regulation). The team observed that insider knowledge, experience and awareness can induce biases, but recognised how these factors also offer richness to interpretations. Moreover, the inclusion of psychologists brought an alternative lens to the analysis.

As our approach to analysis was reflexive, independent coding or inter‐coder reliability was not relevant (Braun and Clarke 2021). Nevertheless, to ensure consistency and relevance, our analysis was sense checked in partnership with stakeholder group members linked to the international ‘Midwives in Focus’ programme hosted by the NNC. Typographical and grammatical errors identified in participant contributions have been corrected to enhance readability.

### 4.7 Data Integration

At the core of mixed‐methods research is the integration of both qualitative and quantitative data, and in convergent designs, the intent is to merge both in order that both quantitative and qualitative results can be leveraged to create a holistic picture in answer to research questions (Fetters, Curry, and Creswell 2013). In this task we took a simultaneous bidirectional approach whereby both the quantitative and qualitative strands are presented separately, and then used to frame the final merging (Moseholm and Fetters 2017). Subsequent to the independent analyses and presentation of data, the results were merged narratively by the research team, using a weaving approach (Fetters, Curry, and Creswell 2013).

### 4.8 Ethical Considerations

The recruitment procedures outlined above began once ethical approval was granted by the lead author's institutional ethics department on the 8 February 2022 (Project reference: P132321). The study was conducted in accordance with the guidelines of good clinical practice. Study participation was voluntary. The data were stored and analysed anonymously. Midwives and nurse‐midwives were only included where they gave informed consent to their participation prior to completing the web‐based survey.

## 5 Results

This study included a total of 860 participants. The top 10 participant countries included the United Kingdom 24% (*n* = 207), Australia 7.8% (*n* = 67), the United States 3.2% (*n* = 28), Kenya 2.8% (*n* = 23), Canada 2.8% (*n* = 24), Saudi Arabia 2.4% (*n* = 21), Uganda 2.2% (*n* = 19), India 2.2% (*n* = 19), Belgium 2% (*n* = 17) and Tanzania 2% (*n* = 17). Representation of midwives (*n* = 443) and nurse‐midwives (*n* = 417) was relatively equal. Statistical analysis could not establish equal representation by country. However, most countries had representation from each profession (e.g., United Kingdom; 186 midwives and 21 nurse‐midwives, Australia; 23 midwives and 44 nurse‐midwives, United States; 3 midwives and 25 nurse‐midwives, Kenya; 1 midwife and 23 nurse‐midwives, Canada; 23 midwives and 1 nurse‐midwife, Saudi Arabia; 4 midwives and 17 nurse‐midwives, Uganda; 10 midwives and 9 nurse‐midwives, Belgium; 12 midwives and 5 nurse‐midwives and Tanzania; 2 midwives and 15 nurse‐midwives). Nevertheless, all 19 participants from India were nurse‐midwives and many countries only had one participant representing them.

Women were strongly represented (93%) alongside 50 men (*n* = 11 midwives; *n* = 39 nurse‐midwives). Two non‐binary participants and a further nine participants who either did not know their gender, preferred not to disclose their gender, or held an alternate identity than those offered also participated in this research. These demographics were considered to be broadly reflective of the general gender distribution in midwifery. The majority of participants (27.0%) were between 35 and 44 years old, 26.0% were between 45 and 54, 19.9% were between 25 and 34 or between 55 and −64, 3.7% were between 18 and 24 and 3.2% were over 65 years of age. In terms of ethnicity and race, 53.8% were White, 23.5% were Black African, 10.3% were Indian Asian, 2.4% were of Hispanic origin and 3.5% were of Middle Eastern or North African descent. The rest of the participants identified as being native/indigenous persons, a Hawaiian/Pacific Islander or ‘other’.

### 5.1 Quantitative Measurement of Professional Identity

In relation to the dimensions of the PIS‐HSP, participants scored highest in ‘professional goals and values’ and in ‘emotional identification and belongingness’, followed by the ‘professional commitment and devotion’ dimension. Participants scored lowest on the ‘self‐fulfilment’ and ‘retention’ dimension (Table 1).

**TABLE 1:** Professional identity subscales for midwives and nurse midwives.

Professional identity subscales	Professional group		Mann Whitney *U*‐test	*z*	Effect size *r*
Dimension	Midwife	Nurse midwife	*p*		
Professional commitment					
Median	7.17	7.67	< 0.001*	−4.12	0.140
25th, 75th percentile	6.17, 8.33	6.67, 8.50			
Minimum, maximum	1, 9	1, 9			
Data available	444	419			
Emotional identity					
Median	7.75	8.00	< 0.001*	−3.38	0.133
25th, 75th percentile	6.50, 8.50	7.00, 8.75			
Minimum, maximum	1, 9	1, 9			
Data available	439	409			
Professional goals					
Median	7.75	8.00	0.042	−2.03	
25th, 75th percentile	7.00, 8.50	7.00, 8.75			
Minimum, maximum	1, 9	1, 9			
Data available	438	407			
Self‐fulfilment					
Median	6.00	7.00	< 0.001*	−5.42	0.187
25th, 75th percentile	4.50, 7.50	5.25, 8.25			
Minimum, maximum	1, 9	1, 9			
Data available	437	407			
Professional identity					
Median	7.14	7.46	< 0.001*	−4.79	0.163
25th, 75th percentile	6.12, 7.92	6.79, 8.31			
Minimum, maximum	2, 9	1, 9			
Data available	445	419			

^*^ indicates that the number is statistically significant.

Overall, nurse‐midwives had a statistically significant higher professional identity score than midwives (*U* = 75,673, *z* = −4.79, *p* < 0.001). The scale factors were separated to explore the specific dimensions of professional identity. For three dimensions, nurse‐midwives had statistically significant higher scores than midwives (Professional Commitment and Devotion, Emotional Identification and Belonging, Self‐Fulfilment and Retention). For one dimension (Professional Goals and Values), the difference between the two groups became non‐significant once the Bonferroni correction was applied. All effect sizes were small. These results indicate that, whilst there is no statistically significant difference in the professional goals and values between single and dual registered participants, dual registered nurse‐midwives self‐report having stronger commitment and belonging in their professional roles. Dual registered nurse‐midwives were also less likely to report a desire to leave their roles or that their self‐worth is negatively impacted by their job.

All dimensions of the scale correlated significantly with each other, as demonstrated by the Spearman correlation coefficient. The Cronbach's alphas for the four dimensions and for the overall scale were 0.852, 0.901, 0.078, 0.803 and 0.916, respectively, which is considered good (Hair et al. 2010), and overlap with the results reported by the original authors of the PIS‐HSP (Liao and Wang 2020).

### 5.2 Qualitative Findings

Themes and subthemes relating to professional identity and representations of midwifery are presented in Table 2. A summary of each is then presented with prose and quotes used to highlight salient meanings and deliver context to the quantitative results offered. Participant quotes are accompanied by a participant number and delineation as to whether the participant was either a Midwife (MW) or Nurse‐Midwife (N‐MW). Not all participants indicated the country in which they were currently working, but geographical locations are presented alongside quotes where available.

**TABLE 2:** Themes and sub‐themes.

Themes	Sub‐themes
1. Reflections on midwives and nurse‐midwives professional identity	Pride and passion in midwifery
	Pride and passion in nurse‐midwifery
	Motivational religiosity
	Celebrating role diversity
	A call for the separation of nursing and midwifery
2. Barriers to midwives' development of a healthy professional identity	Lack of professional recognition in society
	Lack of respect from other professions
3. Reflections on the representation of midwifery	Suggested imagery to represent midwifery
	Suggested mediums to represent midwifery

### 5.3 Theme One: Reflections on Mfidwives' and Nurse‐Midwives' Professional Identity

Overall reflections upon professional identity were grouped into the five subthemes. Participants shared pride and passion in their respective roles, expressed motivational religiosity, celebrated the diversity of their role, and called for midwives independence from nursing.

#### 5.3.1 Pride and Passion in Midwifery

Within this subtheme, many participants broadly described how they ‘love midwifery’ (P73 MW, Uganda) and have a ‘strong passion for midwifery work’ (P38 N‐MW, South Africa). Participants felt ‘proud being a midwife’ (P13 MW), and ‘would not change being a midwife’ (P82 MW, Barbados). In some cases, such pride and passion meant that professional identity merged with one's whole identity.

A midwife is who I am, not just what I do. That will never change. (P205 MW)

I live, breathe, and sweat midwifery. If I had all the money in the world, I would do nothing differently. In my opinion, midwifery is not a profession, it is a way of life. (P39 N‐MW, Israel)

Overly strong professional identities resulted in self‐sacrifice, as one participant described how they ‘would never accept similar working conditions in another role’ and ‘only accept these because [they] love what [their] job should be and caring for those who need [them]’ (P678 MW, United Kingdom). Another described their sacrifice in not ‘having children, even marrying … Every relationship l have ever had has broken down because of reasons relative to my work’ (P689 MW, United Kingdom).

#### 5.3.2 Pride and Passion in Nurse‐Midwifery

Statements under this subtheme relate to pride and passion in nurse‐midwifery. One participant articulates that this particular pride comes from gaining ‘respect’ because of their enhanced ‘knowledge and skills’ (P666 N‐MW, Pakistan).

In contrast to midwives, many nurse‐midwives enjoyed the flexibility of their role. For example, one nurse‐midwife describes how they can put their ‘whole heart in [their] midwifery job and still do other jobs if [they] want’ (P16 N‐MW, Qatar).

I am a proud nurse‐midwife involved in project management. Nurse‐midwives can gain respect because of their knowledge and skills. (P648 N‐MW, Pakistan)

#### 5.3.3 Motivational Religiosity

In this subtheme, several participants described midwifery as their vocational ‘calling’ (P2 N‐MW, Zambia) or ‘“Berufung” (something like a call in German)’ (P163 MW, Germany). One midwife articulates this in the following way: ‘Serving mothers, babies and my community is my calling’ (P186 MW, United Kingdom). Such callings were often linked to religiosity.

I am so passionate about my midwifery profession, I love it 😀, it gives me joy being part of God's agenda. (P243 N‐MW, United Arab Emirates)

I also take midwifery as a vocation from my Christian point of life. (P118 N‐MW, Zambia)

#### 5.3.4 Celebrating Role Diversity

Contributions to this subtheme clearly described the desire for some (predominantly nurse‐midwives) to be seen as more than an expert solely in physiological birth. For example, participants describe their capabilities and successes in other areas and roles such as ‘Business Management’ (P342 N‐MW, Zambia), ‘educator’ (657 N‐MW, Rwanda), ‘academic research’ (P354 N‐MW, Uganda), ‘leadership’ (P121 N‐MW, United Kingdom) and positions ‘further up at the Policy level’ (P711 N‐MW, Czech Republic):

It's not only the job I can do. There are many specialities. (P582 N‐MW, Kenya)

Others described their need to balance their profession with other desires in life:

Whilst midwifery is part of my identity, I am more than just a midwife and I need to be able to have good work‐life balance to be able to be an effective midwife and whole person. (P258 N‐MW, United Kingdom)

Many participants with dual licensure were able to use a greater diversity of skills at work, for example in working as a ‘child health nurse – using [their] midwifery daily’ (P202 N‐MW, Australia).

#### 5.3.5 A Call for the Separation of Nursing and Midwifery

There was a clear call from respondents ‘to see direct entry midwifery across the world. Separate everything from nursing’ (P852 N‐MW, Rwanda). For one participant describing themselves as being in a position of leadership, the conflation between midwifery and the larger profession of nursing meant that they had to remain ‘advocating for recognition of midwifery within the nursing structure’ (P121 N‐MW, Japan). This conflation was highlighted as ‘even the obstetricians call us “the nurses”’ (P826 N‐MW, Australia).

In some geographical areas, being licenced to practise as a nurse‐midwife, rather than being a requirement to practise as a midwife is described as being ‘purely a nursing leadership requirement’ and midwifery was therefore described as being ‘dominated by nursing’ (P188 N‐MW, Australia). To illustrate this, one participant described how they ‘never wanted to be a nurse’, but ‘had to be in order to become a midwife ‐ totally different’ (P839 N‐MW, Canada). In some cases, this was described as a predicament in which potential midwives were discouraged from joining the profession:

My friend never trained as a midwife because she also did not want to have to study nursing first. (P852 N‐MW, Rwanda)

Whilst some expressed how they ‘love being a Nurse and Midwife’ (P601 N‐MW, Australia) independently, others acknowledged and appreciated the complexity of ‘different licensing mechanics’ (P867 MW, United States):

It should be either a nurse or midwife. Can't be both at the same time. Nurse‐midwife is confusing term and should be eliminated. (P495 MW, Saudi Arabia)

### 5.4 Theme Two: Barriers to Midwives' Development of a Healthy Professional Identity

Some participant contributions reflected specifically on barriers to midwives' development of a healthy professional identity and were grouped into subthemes (*n* = 2) accordingly. Such barriers were noted where a lack of professional recognition in society was placed upon the midwifery profession, and a lack of respect from other professions was identified.

#### 5.4.1 Lack of Professional Recognition in Society

Participants described what they perceived to be a lack of professional recognition and investment in midwives' along with their subjugation and devaluation in society. In a context where ‘midwifery is not appreciated by society or government’ (P748 MW, United Kingdom), many called for increased societal recognition in pursuit of a healthier professional identity:

Midwifery work is not valued. I want more from society. (P850 N‐MW, Ethiopia)

Midwifery in Italy is not as respected as it should be … society and the women are not aware about the skills a midwife has. (P753 MW, Italy)

Equally, there was acknowledgement that midwives' professional identity is weakened where the work midwives are expected to do is ‘in opposition to midwifery values’ (P702 MW, United Kingdom).

#### 5.4.2 Lack of Respect From Other Professions

Participants expressed a lack of autonomy and opportunity to embody their professional identity as midwives, as midwifery in some countries (e.g., Latin America) ‘is not well recognized‐ Nor autonomous. [Obstetricians and Gynaecologists] (OBGYNs) decide what to do or how to proceed with low‐risk pregnancy’ (P528 MW, Argentina).

Others articulated challenges in forming a distinct professional identity in midwifery as ‘it is viewed as associate or subservient to obstetrics’ (P32 N‐MW, Lesotho). Similarly, another participant described midwives as being ‘second class citizens to nurses’ (P722 MW, United Kingdom):

I respect my nursing colleagues, but nursing leadership does not respect midwifery and nursing do not want to give up their power. (P188 N‐MW, Australia)

### 5.5 Theme Three: Reflections on the Representation of Midwifery

When asked to reflect on their preferred representations of midwifery within society, diverse participant responses illustrated two subthemes: (1) suggested imagery and (2) suggested mediums.

#### 5.5.1 Suggested Imagery to Represent Midwifery

Many suggested that imagery should showcase midwives in a supportive and leading role during childbirth. One response articulates this image as being ‘Midwife with woman, woman with babe supported by midwife’ (P42 MW, Australia). Imagery containing ‘caring hands’ (P412 MW, United Kingdom) also featured heavily throughout the statements collected, along with birth and midwifery being at the centre of society:

Imagery that demonstrates love, growth and connectivity. (P372 MW, United Kingdom)

Other imagery suggested included ‘butterflies’ (P274 N‐MW, United Kingdom), Midwives depicted as ‘Angels’ (P768 MW, United Arab Emirates), ‘the ocean’ (P729 N‐MW, Somalia) and ‘Onions‐ due to the layers’ (P692 MW, United Kingdom).

“Dawn” ‐ opening scene from lion king film ‐ it epitomises the importance of mother and baby to the family and wider community and in the circle of life. (P691 MW, United Kingdom)

The majority of participants stressed the importance of imagery communicating the ‘role of midwives on the clinical frontline’ (P728 N‐MW, Uganda), using real midwifery ‘characters’ (P607 N‐MW, Nigeria) with ‘personal stories’ (P491 N‐MW, Pakistan).

#### 5.5.2 Suggested Mediums to Represent Midwifery

Sentiments captured within this subtheme describe the mediums participants indicated would best represent the midwifery profession. Arts‐based mediums featured heavily, as participants emphasised the need to have representation of the profession clearly seen within society.

We need something tangible not only digital but other representations through art so the public can see every day. (P43 MW, United Kingdom)

Some participants were keen to engage history and historical artefacts which carry beyond a lifetime in representing the midwifery profession.

Midwives have lost our historic representation because our voices and relics were erased from the historic record. Preserving such items of cultural significance will represent midwifery as a profession in the closest proximity to our actual roles and value as possible. (P410 MW, United States of America)

Several participants also reflected the lack of representation midwives have in the public realm, and suggested mediums with which to overcome this.

We need visuals of midwives, everywhere I see nurses only ‐ can we be recognised in the same way? A statue? (P45 MW, United Kingdom)

We need a midwifery monument ‐ a statue of a midwife rather than a nurse. (P838 N‐MW, United States of America)

Other suggested mediums included public ‘photography and paintings’ (P481 N‐MW, Bermuda), ‘crafts’ (P358 MW, United Kingdom), ‘dance’ (P354 N‐MW, Uganda), ‘tapestry’ (P801 N‐MW, United States of America), ‘song’ (P474 MW, Rwanda), ‘mosaic’ (P823 N‐MW, Australia), ‘crocheting and piano playing’ (P329 MW, United Kingdom), ‘video’ (P258 N‐MW, Iceland), ‘collage’ (P204 MW, United Kingdom) and ‘flags’ (P27 N‐MW, Australia).

There was also a highlighted struggle between representing both the art and science of midwifery, as in contrast to the above, some felt strongly that science should be prioritised over art. For example, one participant wrote ‘knitting does nothing for us from a professional image point of view’ (P667 MW, United Kingdom).

### 5.6 Mixed Methods Interpretations

Quantitative results relating to professional identity highlight that nurse‐midwives had statistically significant higher scores than midwives for three of the four dimensions included in the PIS‐HSP. In terms of professional commitment and devotion, qualitative analyses identified clear commitments to both midwifery and nurse‐midwifery. Yet midwives appeared to express an over devotion to midwifery more than nurse‐midwives did (e.g., making personal sacrifices for midwifery or describing midwifery as ‘a way of life’). Nevertheless, where participants expressed how midwifery was not valued by either society or other professions, it often left them wanting more from their midwifery careers, particularly where they lacked autonomy or opportunities for progression. Describing their motivation to join the midwifery profession as a ‘calling’ and/or for religious reasons also highlighted both midwives' and nurse‐midwives' devotion and commitment to the midwifery profession.

With regards to quantitative emotional identification and belonging scores, qualitative findings revealed clear calls for the separation of midwifery from nursing, particularly where midwifery was seen as being subservient to nursing and/or obstetrics. This desire for the separation was also noted in the suggested imagery to represent midwifery (e.g., a statue of a midwife rather than a nurse). Furthermore, there were identity barriers to overcome in joining the midwifery profession, particularly where there the requirement to register as nurses prior to registering as midwives or ‘nurse‐midwives’ remained, despite these professional groups being viewed as ‘totally different’. In some cases, this conflation resulted in recruitment losses.

Quantitative results in the self‐fulfilment and retention dimension of the PIS‐HSP revealed that midwives were more likely than nurse‐midwives to report a desire to leave their role or that their self‐worth is negatively impacted by their job. In this regard, qualitative findings highlighted how nurse‐midwives or those with the ability to diversify in their roles were better able to thrive overall. Moreover, as midwifery was not seen as being appreciated by society, the public worth of midwives was considered to be lacking overall and many participants called for increased societal recognition in pursuit of a healthier professional identity.

For the PIS‐HSP dimension related to professional goals and values, the statistical difference between the two groups became non‐significant once the Bonferroni correction was applied. Yet dual registered nurse‐midwives self‐report having stronger commitment and belonging in their professional roles and qualitative descriptions highlighted participants having to work in opposition to their midwifery values. They also express further how midwifery is not respected as a profession, either by other professions or society as a whole. In this regard, participants emphasised the need to have representation of the profession clearly seen within society via a variety of different (predominantly arts‐based) mediums. Whilst participants remain aware of the value they bring to society in their midwifery role, in their qualitative responses nurse‐midwives appeared to attribute this more to their advanced knowledge and skills than midwives did.

## 6 Discussion

This study presents foundational insights in relation to professional identity in midwife and nurse‐midwife populations, and how those within the profession perceive they may be best represented in the public realm. The ICM's summaries of both midwifery and nurse‐midwifery in participating countries/regions provide detailed insights about the context of our data collection (UNFPA 2021). Findings offer valuable evidence about professional identity and the importance of the affinity for, acculturation into and identification with the practice of midwifery. Findings also contribute further understandings in relation to how individuals working in dual and single professions structure their identities.

Through a fusion of both our qualitative and quantitative analyses, our mixed‐methods interpretations crucially identify how both the recruitment and retention of midwives is related to lower professional identity scores. Indeed, whilst some participants felt they should qualify as nurses prior to qualifying as midwives, as is the case elsewhere (Mivšek et al. 2015), for many, the prerequisite to become a nurse hindered recruitment to midwifery. This may be because midwives' professional identity can often be a negotiation between medical/pathological models and the philosophy of midwifery (physiological) (Zhang et al. 2021). Midwives scores in relation to both commitment/devotion and self‐fulfilment/retention were also linked to a lack of autonomy and recognition from both society and other professional groups. Elsewhere, intrinsic factors such as lack of autonomy and recognition have similarly been identified as the main factors related to midwife burnout (Andina‐Díaz et al. 2024). As such it will be important to strengthen the professional identity of midwives in pursuit of improved recruitment and retention within the profession, particularly in light of the World Health Organisation's new global position statement on transitioning to midwifery models of care worldwide (WHO 2024). Given the interpretations presented here, midwives' enhanced recognition, respect and professional identity may usefully be achieved via public arts‐based installations (e.g., monuments). The separation of midwifery from nursing may also enhance the professional identity of midwives, particularly through the emotional identification and belonging dimension. This shift may also enhance the profile and autonomy of the profession as midwives may no longer be seen as subservient to nursing and/or obstetrics. Challenges in relation to some midwives' overly strong (e.g., unhealthy) professional identities remain. Increasing the diversity of the midwifery role (e.g., in research, education, policy and leadership) may go some way toward challenging perceptions in this regard as well as opening up new possibilities for career progression.

Linked to expressions of service and sacrifice, the evidence presented in relation to an overly strong (rather than healthy) professional identity in some participants is concerning, as these can lead to poor teamwork, resistance to change, incivility and bullying (Stevens 2013). These findings align with those published elsewhere (Bloxsome, Bayes, and Ireson 2020). Though such excessive endurance in midwifery in particular is seen to result in burnout and is highlighted for example where the ‘good midwife’ is identified as someone whose practice goes beyond what is actually required (Carolan 2013). Over‐identification with one's professional identity can also supersede one's sense of personal self, accentuating tensions in this regard (Kwok and Sumser 2024). Rigid ideas about one's professional identity can similarly thwart innovations and improvements in healthcare (Powell and Davies 2012). Where participants had been motivated by religion to practise midwifery, sometimes describing this as a ‘calling’, they may also attribute their work to divine meaning, permeated by thoughts associated with a spiritual propensity (Begnini et al. 2021). Yet both historical and contemporary images of nurses and midwives marked by angelical and heroic figures and strongly influenced by religiosity can problematically feminise and undermine the expertise of the workforce (Stokes‐Parish et al. 2020). Thus, the development of future professional identities in midwifery may be usefully reimagined away from religiosity, ridged ideas and excessive endurance toward a more progressive and adaptable opportunity to instead frame professional identity around excellence in perinatal health, wellbeing and outcomes.

Weaker professional identities are associated with poorer standards of care (Traynor and Buus 2016) along with poorer retention rates and inadequate teamwork (Koh et al. 2023). Increased rates of attrition can also be attributable to discrepancies between idealised professional identities and disillusioned ones (Carolan 2013; Traynor and Buus 2016). This may not be surprising in midwifery, where despite being prepared for autonomous practice and embracing the philosophy of the profession as students, midwives are often stifled and denied autonomy once established in some areas (Kraienhemke 2021; Small et al. 2022). Moreover, celebrated role diversity may be protective against attrition (Caza and Creary 2016) and may be realised more broadly in developing advanced midwife practitioners (Goemaes et al. 2016, 2018). As has been found elsewhere (Caza and Creary 2016), many nurse‐midwife participants in our study applied their skills both as midwives and nurses across situations. Nevertheless, it will be important to ensure that people with dual professional roles also feel part of all their distinct professional communities (Best and Williams 2019). Future research could usefully explore whether and how regional organisations achieve this (or not).

Though both nurses and midwives play key roles in the promotion of health and the prevention of illness, midwifery aligns with a holistic model of care in the context of childbearing (a physiological event), while nursing incorporates various models pertaining largely to ill health. Therefore, both nursing and midwifery skills can become obsolete in these opposing domains, and role confusion can result in conflict and poor clinical care (Jayathilake et al. 2021). Ultimately, nurse‐midwives perceive themselves to be nurses, regardless of where they practise (e.g., in perinatal services), which is oftentimes viewed as unproblematic (Nicacio et al. 2016). Yet clear distinctions not only help the public understand unique roles and expertise, enabling them to make informed choices about their care (Callister 2020), they also enhance professional accountability as each profession is responsible for adhering to its own standards of practice and ethical codes (Doherty 2020), facilitating effective teamwork by clarifying roles and responsibilities (Reeves et al. 2011), leading to better patient outcomes overall. The conflation noted within these findings was highlighted as being problematic in several ways. Therefore, the separation of midwifery from nursing may offer new opportunities for strengthening midwives' professional and public identity along with their recruitment and retention worldwide.

Public artworks (e.g., statues) can act as collective memory anchors for professions, linking practitioners to their historical roots and reinforcing a sense of shared professional identity (Gongaware 2010). Indeed, by commemorating pioneers, leaders and significant achievements, these can contribute to a profession's collective memory and foster a sense of continuity and belonging among its members, serving as visual reminders of a profession's values and ideals. Presently in the public realm, representations of midwifery are observably scarce and predominantly drawn from fiction, while nursing is represented principally by revered historical characters (e.g., Florence Nightingale), some of whom are also drawn upon to represent midwives (Pezaro, Maher, and Fissell 2022). This is perhaps reflective of how the nursing profession has historically been perceived as being better educated and thus superior (Luyben et al. 2017). Nursing may also have been given precedence over midwifery due to nurses' integration into the medical model, where professional hierarchies often reflect and reinforce gendered stereotypes and power structures (Witz 2013). Given the need to enhance midwives' professional and public identity, future representations of them via public artworks are urgently required and may usefully be based upon both historical and contemporary realities (Madsen et al. 2009; Pezaro, Maher, and Fissell 2022).

Whilst many participants suggested representing midwifery in proximity to childbirth via arts‐based approaches, this was somewhat at odds with other participants' desire to be respected in areas of practice outside of the clinical realm (e.g., research). Such qualitative findings may allude to a tension between celebrating both the ‘art and science’ of midwifery in which some midwives seek to portray themselves as vessels of service and sacrifice and/or religious characters (e.g., angels) feminised in art, and yet simultaneously call alongside others to be recognised, valued and respected firmly in science. Many other highly gendered images were also suggested by participants to represent them (e.g., women being with women). This may not be surprising in a context where professional identity construction may be shaped by societal stereotypes about gender (Bian, Leslie, and Cimpian 2017). Yet this may hinder progress toward reproductive justice and improved perinatal outcomes, particularly where white cisheteropatriarchal norms prevail and remain oppressive in the perinatal space (Pezaro et al. 2023; Pezaro, Pendleton et al. 2024). Consequently, future representations of midwifery may usefully be progressive in moving away from stereotypes, whilst delicately balancing both the art and science of the profession in this context.

Our findings remain congruent with the World Health Organization's global report (WHO 2016), where midwives similarly reported a lack of respect from colleagues, the community and wider society. Lower levels of professional identity and societal recognition for midwives are associated with poorer standards of care (Traynor and Buus 2016) and professional burnout (Andina‐Díaz et al. 2024) along with poorer recruitment and retention. In a context where the world is transitioning to midwifery models of care (WHO 2024) in order to reduce the majority of preventable perinatal deaths worldwide (Renfrew 2021), action to enhance the professional identity of midwives is urgently required. A person's authentication emerges as a socially constructed process of both determining who one is and helping others see who one is (Caza, Moss, and Vough 2018). Consequently, in line with the findings presented here, it would be prudent to embody the midwifery profession through public artworks to promote their societal and professional recognition and value, which in turn may lead to increased autonomy and influence for the profession in optimising perinatal outcomes worldwide. Efforts in this area have already begun via www.findthemidwife.com.

### 6.1 Limitations and Directions for Future Research

Whilst this study is the first of its kind and is a progressive step forward in global research beyond anglophone countries, the nature of data collection, relatively low sample size and our study design limits conclusions on causality of the differences between identity construction. Additionally, it is not possible from our data to identify antecedents to identity construction and related outcomes in these occupational groups in order to recommend clearer actions for practice, particularly with such a diversity of contexts. Indeed, we recognise that the very different working circumstances and role perceptions (Carvajal et al. 2024), along with public, cultural and societal differences may have shaped the data analysis in this regard. Nevertheless, the purpose of this research was not to achieve absolute representative samples from each country but to gather responses from as many countries as possible to give voice to midwives practising across different parts of the world.

Our mixed‐methods approach is a key strength which enabled us to enhance the interpretation of our data overall. For example, on their own, some of the qualitative findings suggest that both midwives and nurse‐midwives may have equally strong professional identities. However, the quantitative results evidence that this is not the case overall. We may have strengthened our triangulations further by collecting quantitative data in relation to the third research aim.

Inadequate diversity of participants prevented further analysis and limited the generalisability of our findings. Nevertheless, we observed a trend of increasing overall identity score with age, particularly in relation to the self‐fulfilment dimension. Future research could assist in identifying why some midwives persist in their profession despite describing toxic work environments and explore institutional steps that are needed to protect their retention. Deeper exploration of how different models of regulation and professional identity affect the quality of care, patient outcomes and job satisfaction among both nurse‐midwives and midwives may also be useful in unifying shared visions for the future. Equally, the variation of midwifery perceptions across geographical areas indicates a need for further localised practice recommendations which also consider how institutions might ensure equal representation between professional groups. Essentially the impetus now is to explore potential differences across countries, languages and cultures.

## 7 Conclusion

This is the first study to both measure and investigate the professional identity of midwives and nurse‐midwives concomitantly and explore how midwifery may be best represented in the public realm in pursuit of midwives enhanced professional identity and recognition in society. In our sample, nurse‐midwives had higher levels of professional identity than midwives. This may be due to a number of factors such as the absence of a unifying Midwifery identity rooted in shared history, and the misrepresentation of midwives through mythological figures. Whilst there are clear calls to separate the midwifery and nursing professions, the increased complexity of roles may provide opportunities for diversity in future workforce planning and be protective against workforce attrition. Societal representations of midwifery (e.g., statues) which balance both the art and science of midwifery in line with the level presently afforded to nursing are required to support the development of healthier professional identities and enhance recruitment, retention and perinatal outcomes overall.

## Conflicts of Interest

The authors declare no conflicts of interest.

### Peer Review

The peer review history for this article is available at https://www.webofscience.com/api/gateway/wos/peer‐review/10.1111/jan.16696.

## Supporting information


Data S1.

## Data Availability

The data that support the findings of this study are either available within this article, or available on request from the corresponding author (S.P.). The data are not all publicly available due to their containing information that could compromise the privacy of research participants.
