# Supplementary material for: Exploring Midwives' and Nurse‐Midwives' Professional Identity and How Midwifery May Be Best Represented in the Public Realm: A Global Convergent Parallel Mixed‐Methods Study
Source: J Adv Nurs. 2024 Dec 26;81(6):3283–95. doi: 10.1111/jan.16696 (PMC12080075; doi:10.1111/jan.16696)
Supplement: Supplementary file 1 — Data S1. [file JAN-81-3283-s001.docx]

**Good Reporting of A Mixed Methods Study (GRAMMS)**

| **Guideline** | **Section: page** |
| --- | --- |
| Describe the justification for using a mixed methods approach to the research question | p4 |
| Describe the design in terms of the purpose, priority and sequence of methods | p4-5 |
| Describe each method in terms of sampling, data collection and analysis | p5-7 |
| Describe where integration has occurred, how it has occurred and who has participated in it | p7 |
| Describe any limitation of one method associated with the present of the other method | P20 |
| Describe any insights gained from mixing or integrating methods | p20 |

*O'Cathain A, Murphy E, Nicholl J. The quality of mixed methods studies in health services research. J Health Serv Res Policy. 2008;13(2):92-98.*
